# Our Covid odyssey

**DOI:** 10.1177/10398562231164868

**Published:** 2023-03-24

**Authors:** George Halasz

**Affiliations:** Clayton, VIC

During Covid isolation a word, unwholesome pierced my heart’s dolesome beats lament     lament    lament

Beyond simple grief’s silent wail behind bitter sweet ruptures, betrayal after betrayal lament     lament    lament

Past plaintive isolation I plunged into plangent alienation lament     lament    lament

Fits of privation brought to mind access to a familiar family refrain to reawaken still a deeper isolation

How insane, this internal collapse

My hopeless independence, scorched self-alienation helplessly dependent on privation’s double subjugation

With each lament, I untangled Penelope’s shroud: boredom, fury, tedium, rage and vengeance

From finality’s aura, to reclaim golden threads of reality renewing a silver lining, our elusive inter-dependence

We made peace, reclaimed our individual agency, freedom simply to be, you and me

